# Superior vena cava and atrial cardiomyopathy: role of adjunctive superior vena cava isolation ablation in atrial fibrillation

**DOI:** 10.1093/europace/euaf204

**Published:** 2025-09-02

**Authors:** Bharat K Kantharia, Xander H T Wehrens, Dominik Linz

**Affiliations:** Division of Cardiac Electrophysiology, Cardiovascular and Heart Rhythm Consultants, 30 West 60th Street, Suite 1U, New York, NY 10023, USA; Division of Cardiology, Mount Sinai Morningside-St Luke's Hospital, Icahn School of Medicine at Mount Sinai, 1111 Amsterdam Avenue, New York, NY 110023, USA; Cardiovascular Research Institute, Departments of Integrative Physiology and Medicine, Baylor College of Medicine, Houston, TX, USA; Division of Cardiac Electrophysiology, Department of Cardiology, Maastricht University Medical Center, Maastricht, The Netherlands; Department of Biomedical Sciences, Faculty of Health and Medical Sciences, University of Copenhagen, Copenhagen, Denmark

**This editorial refers to ‘Pulmonary vein isolation with or without empiric superior vena cava isolation in patients undergoing ablation for paroxysmal atrial fibrillation: the randomized ESVCI-AF trial’ by W. Shen *et al.*, https://doi.org/10.1093/europace/euaf175.**

Atrial cardiomyopathy, through structural, architectural, contractile, or electrophysiological alterations, affects the electrochemical coupling function of cardiomyocytes, resulting in focal enhanced automaticity, triggered activity, and/or micro re-entry from the myocardial tissue throughout the atrium, which leads to arrhythmogenesis.^1^

The substrate of atrial fibrillation (AF) may involve more than pulmonary vein (PV) triggers. Extra-PV sites that could serve as potential AF triggers have an incidence of 28%, and among non-PV sites, the incidence arising from the superior vena cava (SVC) is about 37%.^2^ Despite differences in their embryological origins, SVC and PVs share several common features. Atrial myocardial sleeves extending into the vein structures, ‘myocardium meets vessel’, are seen in both SVC and PVs. In humans, SVC-atrial myocardial extension sleeve is short and solely sub-epicardial with myocardial fibres in the sleeves mostly organized in a circular orientation relative to the vein’s long axis and often exhibiting discontinuity.^2^ Along with the sinus node and right phrenic nerve, the SVC bears an important spatial relationship with the right superior pulmonary vein (RSPV) both being adjacent to each other^2^ (*Figure 1A*). In arrhythmogenic SVC, effective refractory period (ERP), a metric linked to atrial cardiomyopathy, is significantly shorter.^3^ Degenerative changes with fibrosis have also been described in approximately one-third of all myocardial extensions of PVs and SVC, making them vulnerable to various atrial arrhythmias including AF.^1,2^

**Figure 1:**
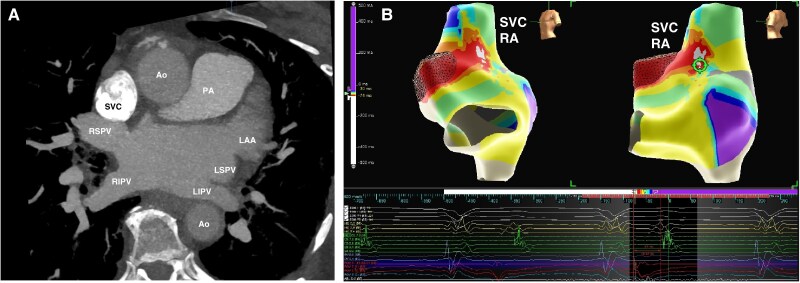
(*A*) Transverse axis view of cardiac CT shows contrast filled superior vena cava that is situated adjacently anterior and superior to the right superior pulmonary vein. Other labelled structures are the right inferior pulmonary vein (RIPV) on the right, the left superior pulmonary vein, the left inferior pulmonary vein, and the left atrial appendage on the left, aorta and pulmonary artery anteriorly, and descending aorta posterior to the left inferior pulmonary vein. (*B*) Electroanatomic (Ensite NavX, Abbott Cardiovascular, USA) local activation map of atrial tachycardia (cycle length 350 ms), colour coded for early to late activation sites. Atrial tachycardia confined to the superior vena cava-right atrial junction was ablated terminating the tachycardia and isolating superior vena cava. Ao, aorta; CT, computed tomography; LAA, left atrial appendage; LIPV, left inferior pulmonary vein; LSPV, left superior pulmonary vein; PA, pulmonary artery; RA, right atrial; RIPV, right inferior pulmonary vein; RSPV, right superior pulmonary vein; SVC, superior vena cava.

When may arrhythmogenicity of SVC be suspected during catheter ablation of AF, with a view to pursue SVC isolation (SVCI)? Yoshida *et al.*,^4^ suggested SVCI to be considered in the following scenarios; (i) when ectopic beats from the SVC are observed with or without the initiation of AF; (ii) rapid activities (cycle length < 120 ms), fractionated or continuous electrograms, or double potentials are observed continuously or intermittently in the SVC during tachycardia; and (iii) after PV isolation (PVI), a sustained tachycardia is observed in the SVC despite the restoration of sinus rhythm in the atria. More commonly, arrhythmogenicity of the SVC as part of SVC-atrial cardiomyopathy may manifest in the form of short atrial ERP and development of SVC-right atrial junction atrial tachycardia either spontaneously or with pharmacological and pacing provocation during AF ablation, as was in the case of a 74-year-old woman with prior failed cardioversion, antiarrhythmic drug refractory persistent AF, and evidence of burst of atrial tachycardia and AF on non-invasive rhythm monitoring who underwent ablation (*Figure 1B*).

A close link between SVC arrhythmogenesis and arrhythmogenesis in the RSPV has been observed. In a study by Yoshida *et al.*,^4^ the prevalence of arrhythmogenic SVC was 32% when the RSPV was arrhythmogenic during PVI ablation. The sensitivity, speciﬁcity, and positive and negative predictive values of RSPV arrhythmogenicity for predicting SVC arrhythmogenicity were 86%, 59%, 32%, and 95%, respectively.^4^ While SVC arrhythmogenicity was widely observed in women and individuals with smaller body surface area, in a multivariable logistic regression analysis, arrhythmogenic RSPV was the only independent predictor of arrhythmogenicity of the SVC [odds ratio (OR), 8.53; 95% confidence interval (CI), 2.31–31.46; *P* = 0.001].^4^

An arrhythmogenic SVC was detected in 5.3% of cohort of 836 patients, predominantly after the first catheter ablation procedure in a study by Miyazaki *et al*.;^5^ Identification of an arrhythmogenic SVC occurred with spontaneous or isoproterenol challenge in 56.8%, adenosine challenge in 29.5%, and pacing stimulation in 13.6%. These patients were younger and less obese and had smaller left atria and presented predominantly with paroxysmal AF and concurrent common atrial flutter (AFL) compared to those without SVC arrhythmogenicity. A multiple logistic regression analysis revealed that the left atrial (LA) size (OR, 0.93; 95% CI, 0.88–0.99) and coexistence of AFL (OR, 2.01; 95% CI, 1.00–4.02) were independent predictors identifying an arrhythmogenic SVC. A meta-analysis examining the clinical and genetic characteristics of 2170 AF patients, both with and without SVC arrhythmogenicity, revealed that lower LA dimensions and the rs2634073 and rs6584555 single-nucleotide polymorphism genotypes were linked to SVC arrhythmogenicity.^6^ Furthermore, co-variables that were conditionally associated with SVC arrhythmogenicity included younger age, lower body mass index, and higher left ventricular ejection fraction.^6^

Superior vena cava isolation is routinely performed during surgical Cox-Maze and hybrid surgical and catheter ablation procedure for AF whether performed as *de novo* surgery/procedure for AF or with concomitant cardiac surgeries.^7^ As a catheter-based procedure, it is feasible to perform SVCI successfully by radiofrequency (RF) ablation at conventional power and temperature or very high-power short duration (vHPSD) settings, cryoablation with cryoballoon technology, and even pulse field ablation (PFA); a rapidly emerging preferred method of ablation due to its novel electroporation mechanism of inducing cell necrosis and deeper tissue selective lesions.^8–10^ The crucial question, however, persists as to whether ablation comprising SVCI, as an adjunct to PVI, would enhance long-term freedom from AF in patients with various forms of AF, be as an initial treatment or during a re-intervention following recurrence and across diverse phenotypes of atrial cardiomyopathy.

In the current issue of *EP Europace*, Shen *et al.*,^11^ present the results of their prospective, multicentre, randomized trial of empiric SVCI combined with PVI (ESVCI-AF) in patients with paroxysmal AF undergoing first-time catheter ablation. A total of 302 patients (median age 64.9 years, 54.6% male) were randomized to receive either PVI combined with SVCI or PVI alone. Ablation index-guided lesions were administered utilizing a three-dimensional electroanatomical mapping system and irrigated-tip, contact force RF catheters. The procedural endpoints were the achievement of a bidirectional conduction block in the PVs and SVC. Pharmacological provocation with isoproterenol or adenosine after ablation was not conducted. Following a median follow-up of 20 months, the recurrence rate of atrial tachyarrhythmias in the absence of antiarrhythmic drugs was statistically comparable: 13.2% in the PVI plus SVCI cohort and 19.2% in the PVI-only group [hazard ratio (HR), 0.68; 95% CI, 0.38–1.20], with a trend towards reduced AF recurrence in females (29.7% vs. 14.3%; HR, 0.47; 95% CI, 0.21–1.02; *P* = 0.055).^11^ There were no instances of sinus node injury with SVCI, and reversible phrenic nerve injury was reported in only one patient (0.6%).^11^

The study had a relatively small cohort size; continuous rhythm monitoring to determine AF burden and ‘shorter/asymptomatic’ AF episodes was not utilized; and patients with persistent AF were excluded. These limitations affect the overall interpretation and extrapolation of the study findings. Moreover, it remains ambiguous how many patients in the PVI plus SVCI group required a repeat ablation procedure, and if applicable, whether the SVC remained persistently isolated or not.^11^

In another study by Dong *et al.*,^12^ which evaluated SVCI alongside PVI for paroxysmal AF, published not too long ago in *EP Europace*, the patients underwent a stringent provocation protocol that included a pharmacological challenge and multisite burst atrial pacing to assess for SVC arrhythmogenesis. If SVC triggers were detected, strategy of SVCI with PVI ablation and additional non-PV foci was used in these patients. This protocol-based SVCI failed to demonstrate better freedom from atrial tachyarrhythmias at 12 months (87.9% with SVCI vs. 79.6% without SVCI).^12^ Interestingly, in a randomized study of by Corrado *et al.*^13^, SVCI in conjunction with PVI ablation yielded a 12-month procedural successful outcome exclusively in patients with paroxysmal AF and not in those with non-paroxysmal, i.e. persistent and permanent, AF.

As for ablation with cryoballoon technology, in the CAVAC AF (Superior Vena Cava isolation by Cryoballoon in Addition to Pulmonary Vein Isolation in Atrial Fibrillation Ablation Patients) trial, the combination of SVCI with PVI did not yield superior freedom from AF recurrence compared to PVI alone (62.9% vs. 72%) in patients with paroxysmal or non-long-standing persistent AF between 91 and 365 days post-catheter ablation. Furthermore, SVCI was associated with significantly higher rates of phrenic nerve paralysis (20.8% vs. 6%) and transient sinus node injury (18.8% vs. 0%) compared to PVI alone.^9^

A recent meta-analysis of four randomized controlled trials involving catheter ablation for paroxysmal AF, comprising 287 patients undergoing PVI plus SVCI and 313 patients receiving PVI alone, demonstrated a significant reduction in AF recurrence with SVCI plus PVI (11.7%) compared to PVI alone (19.9%).^14^ Nonetheless, as frequently observed in meta-analysis methodology, there was significant residual heterogeneity, confounding the synthesis of evidence with this meta-analysis.

It is worth noting that empiric adjunct ablation strategies on top of PVI are often ineffective. In CAPLA (Catheter Ablation for Persistent Atrial Fibrillation: A Multicentre Randomized Trial of Pulmonary Vein Isolation vs. PVI With Posterior Left Atrial Wall Isolation) study, ‘empiric’ LA posterior wall isolation failed to reduce AF recurrences in patients with persistent AF undergoing first-time catheter ablation.^15^ In clinical practice, more extra-PV sites are targeted during catheter ablation of persistent AF as conceptually greater degree of atrial cardiomyopathy is considered to be present in persistent AF. At least for ablation for paroxysmal AF, along with several studies conducted in the past, the current commendable study by Shen *et al.*,^11^ which showed the ineffectiveness of routine ‘empiric’ SVCI alongside PVI, the debate of utility of ‘empiric’ SVCI as an adjunct to PVI may be put to rest. Nonetheless, the significance of SVCI must be assessed by future larger studies across specific subgroups, including gender, comorbidities, different forms of AF, and different morphologies and morphometries of the SVC and LA. Also, more basic science and translational research related to the relationship between SVC and atrial cardiomyopathy must be encouraged.

## Authors’ contribution

B.K.K.: conceptualization, resources, writing—original draft, writing—review & editing. X.H.T.W. and D.L.: writing—review & editing.

## Data Availability

No new data were generated or analysed in support of this research.
